# Three-year outcomes of the TCD-17187 (Kanshas) drug-coated balloon for the treatment of atherosclerotic lesions in the superficial femoral and proximal popliteal artery

**DOI:** 10.1186/s42155-026-00695-7

**Published:** 2026-04-24

**Authors:** Shu-Ichi Seki, Yoshimitsu Soga, Osamu Iida, Daizo Kawasaki, Hitoshi Anzai, Hiroshi Ando, Tatsuya Nakama, Norihiko Shinozaki, Amane Kozuki, Masaharu Ishihara, Kazushi Urasawa, Fumiyuki Hayashi, Hiroaki Tsujita, Kazuki Tobita, Kenji Ogata, Kazunori Horie, Naoki Hayakawa, Shinsuke Mori, Masahiko Fujihara, Takao Ohki, Kenichiro Yuba, Toshiaki Mano, Masato Nakamura

**Affiliations:** 1 Department of Medicine and Cardiology, Chikamori Hospital, Kochi, Japan; 2 Department of Cardiology, Kokura Memorial Hospital, Fukuoka, Japan; 3 Division of Cardiology, Cardiovascular Center, Osaka Police Hospital, Osaka, Japan; 4 Department of Cardiology, Morinomiya Hospital, Osaka, Japan; 5 Department of Cardiology, SUBARU Health Insurance Ota Memorial Hospital, Gunma, Japan; 6 Heart Center, Kasukabe Chuo General Hospital, Saitama, Japan; 7 Department of Cardiology, Tokyo Bay Medical Center, Chiba, Japan; 8 Department of Cardiology, Tokai University Hospital, Kanagawa, Japan; 9 Division of Cardiology, Osaka Saiseikai Nakatsu Hospital, Osaka, Japan; 10 Department of Cardiovascular and Renal Medicine, School of Medicine, Hyogo Medical University, Hyogo, Japan; 11 Department of Cardiology, Caress Memorial Hospital, Hokkaido, Japan; 12 Division of Minimally Invasive Treatment in Cardiovascular Medicine, Toho University Ohashi Medical Center, Tokyo, Japan; 13 Division of Cardiology, Department of Medicine, Showa University School of Medicine, Tokyo, Japan; 14 Cardiology and Catheterization Laboratories, Shonan Kamakura General Hospital, Kanagawa, Japan; 15 Department of Cardiology, Miyazaki Medical Association Hospital, Miyazaki, Japan; 16 Department of Cardiovascular Medicine, Sendai Kousei Hospital, Miyagi, Japan; 17 Department of Cardiovascular Medicine, Asahi General Hospital, Chiba, Japan; 18 Department of Cardiology, Saiseikai Yokohama City Eastern Hospital, Kanagawa, Japan; 19 Department of Cardiology, Nozaki Tokushukai Hospital, Osaka, Japan; 20 Division of Vascular Surgery, Department of Surgery, Jikei University School of Medicine, Tokyo, Japan; 21 Department of Cardiology, Tokushima Red Cross Hospital, Tokushima, Japan; 22 Cardiovascular Center, Kansai Rosai Hospital, Hyogo, Japan

**Keywords:** Peripheral artery disease, Drug-coated balloon, Superficial femoral artery, Popliteal artery, Primary patency, Clinical outcomes

## Abstract

**Background:**

This study aimed to evaluate the safety and effectiveness of the Kanshas drug-coated balloon (DCB) with paclitaxel for the treatment of atherosclerotic lesions in the superficial femoral artery (SFA) and/or proximal popliteal artery (PA) over a 3-year period.

**Results:**

A prospective, multicenter, single-arm trial enrolled 121 patients with symptomatic lower extremity artery disease (LEAD). At 3 years, the primary patency rate was 63.4%, and freedom from clinically driven target lesion revascularization (CD-TLR) was 83.2%. Sustained improvements were observed in Rutherford classification, ankle brachial index (ABI), and walking impairment questionnaire (WIQ) scores. No device- or procedure-related deaths or major amputations occurred.

**Conclusions:**

The Kanshas DCB showed favorable safety and effectiveness for treating atherosclerotic lesions in the SFA and/or proximal PA over 3 years.

**Trail registration:**

Registration ID: UMIN000034122. Registration Date: September 13, 2018. Registration site URL: https://center6.umin.ac.jp/cgi-openbin/ctr/ctr.cgi?function=brows&action=brows&recptno=R000038612&type=summary&language=J.

**Graphical Abstract:**

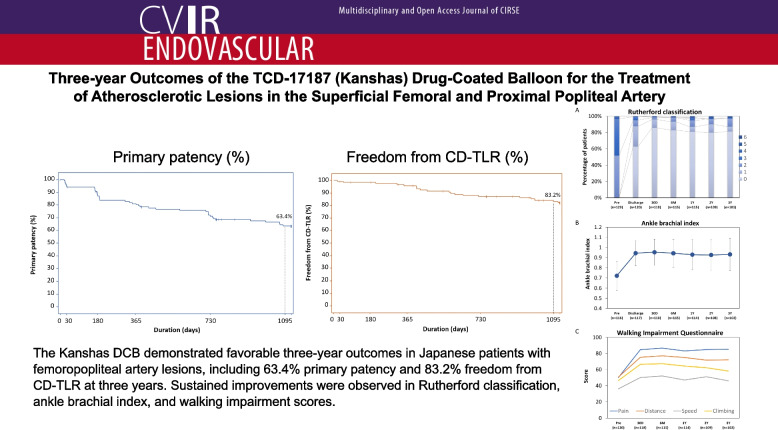

**Supplementary Information:**

The online version contains supplementary material available at 10.1186/s42155-026-00695-7.

## Background

The Kanshas DCB uses “Unicoat” technology, which uniformly coats the balloon surface with 3.2 μg/mm^2^ of paclitaxel microcrystals and the carrier, L-SEE, to facilitate drug transfer to the arterial wall and minimize downstream effects [1]. Previous publications documented the 12- and 24-month outcomes of the Kanshas DCB in a Japanese patient population [2, 3]. In these reports, the primary patency rates at 12 and 24 months were 81.1% and 71.3%, the freedom of clinically driven target lesion revascularization (CD-TLR) rates were 95.8% and 87.0%, and the major adverse event (MAE) rates were 5.0% and 13.2%, respectively. The study revealed a total absence of fatalities or major amputations that were associated with the use of the device or the implementation of the procedure. These results were clinically satisfactory. This document presents the conclusions of a 3-year study that sought to assess the safety and effectiveness of the Kanshas DCB in the treatment of atherosclerotic lesions of the superficial femoral artery (SFA) and/or the proximal popliteal artery (PA).

## Methods

### Study design

This was a prospective, multicenter, core laboratory-adjudicated, single-arm trial conducted at 21 centers in Japan between October 2019 and November 2020. Prior to participant enrollment, written informed consent was obtained from all patients in accordance with protocols approved by the institutional review boards (IRBs) at each investigational site. The detailed participant inclusion and exclusion criteria are delineated in the supplementary appendices of the 1-year and 2-year reports [2, 3]. Procedures were performed under standard antiplatelet therapy. Pre-dilatation was mandatory, but the use of special PTA (percutaneous transluminal angioplasty) balloons, such as cutting or scoring balloons was not permitted as this study used the results of previously conducted PTA as the performance goal. The DCB size was matched to the reference vessel diameter in a 1:1 balloon-to-vessel ratio with a recommended inflation time of at least 180 s. Post-dilatation with a standard PTA balloon was allowed at the operator’s discretion. Provisional stenting was permitted only in cases of PTA failure after repeated and prolonged balloon inflations, defined as residual stenosis of ≥ 50% or major flow-limiting dissection (≥ Grade D). DAPT was continued for at least 1 month following the index procedure. Follow-up assessments were conducted at 30 days, 6 months, 1 year, and 2 years post-procedure, with the final follow-up planned for 3 years postoperatively. At each follow-up, we assessed using DUS, and any major adverse events (MAE) and other adverse events were recorded. The primary endpoint was primary patency (freedom from restenosis and CD-TLR by duplex ultrasound), and the safety endpoint was major adverse events (MAE: 30-day death, major amputation, or CD-TLR). Statistical analyses included Kaplan–Meier estimates and Cox regression analysis. The study was independently monitored by a data safety monitoring board (DSMB) and clinical events committee (CEC), which reviewed and adjudicated all adverse events over the 3-year period following DCB treatment. Independent core laboratories analyzed procedural and follow-up images. The study adhered to the Declaration of Helsinki, good clinical practice guidelines, and all applicable laws as specified by relevant governmental authorities.

### Study population

Patients with symptoms of claudication and/or ischemic rest pain (Rutherford classification 2–4) due to stenotic or non-stented restenotic lesions with a total lesion length of ≤ 18 cm or totally occlusive lesions with a length of ≤ 10 cm involving the SFA and proximal PA, were eligible. The proximal PA included the area from the adductor hiatus (descending genicular artery) to the superior border of the patella (P1).

### Description of study device

The study device utilized the Kanshas DCB (Terumo, Tokyo, Japan), which employs “Unicoat” technology, featuring a carrier containing 3.2 μg/mm^2^ of paclitaxel microcrystals and L-SEE. The balloon is uniformly coated with paclitaxel microcrystals, which facilitates drug delivery to the arterial wall and minimizes downstream effects [1].

### Definitions

Primary patency was defined as a core laboratory-assessed DUS peak systolic velocity ratio of less than 2.4 in the absence of CD-TLR. CD-TLR was defined as re-intervention at the target lesion due to the recurrence of symptoms with ≥ 50% restenosis by core laboratory assessment, worsening of Rutherford classification, or a decrease in ABI of ≥ 0.15, when compared to the post-procedure baseline. Major amputation was defined as the amputation of the limb above the ankle. Technical success was defined as residual stenosis in the treated segment of 30% or less, without grade D or greater vessel dissection. Procedural success was defined as achieving technical success without the occurrence of a major adverse event (MAE) during the index procedure. An MAE was defined as a composite of device- and procedure-related 30-day death, or an index limb major amputation and/or CD-TLR during follow-up.

### Study outcome measures

The primary effectiveness and safety endpoints were previously reported through 12 and 24 months [2, 3]. The primary efficacy outcome measure was primary patency after the index procedure up to 3 years. The secondary efficacy outcomes included (1) freedom from CD-TLR, (2) change in ABI, (3) clinical improvement based on the Rutherford classification, and (4) the Walking Impairment Questionnaire (WIQ) score throughout the follow-up period. The primary safety outcome measure was the MAE rate. MAEs were evaluated at 30 days, 6 months, 1 year, 2 years and 3 years. Death, repeat revascularization, and amputation were independently adjudicated by the CEC.

### Statistical analysis

The Kaplan–Meier method was employed to evaluate time-to-event data for primary patency at the 3-year follow-up. In the exploratory data analysis, the hazard ratio was calculated using a univariate Cox regression model to examine patient, lesion, and procedure characteristics that may affect primary patency. For the multivariate analysis with the Cox regression model, backward elimination was used with an elimination criterion of a *p* value ≤ 0.1. Continuous variables were expressed as mean ± standard deviation, while categorical variables were presented as counts and proportions. Statistical analyses were performed using SAS software version 9.4 (SAS Institute, Cary, NC, USA), except for Figure S1, which was analyzed using R software version 4.2.2 (R Foundation for Statistical Computing, Vienna, Austria).

## Results

The trial enrolled 121 symptomatic LEAD patients with SFA and/or proximal PA lesions.

### Baseline patient and lesion characteristics

It is imperative to acknowledge that baseline and procedural characteristics are not delineated in this report, as these have previously been expounded in the 12- and 24-month reports [2, 3]. Comprehensive tabular data are provided in Tables S1 and S2 of the supplemental material.

### Efficacy outcomes

**Fig. 1:**
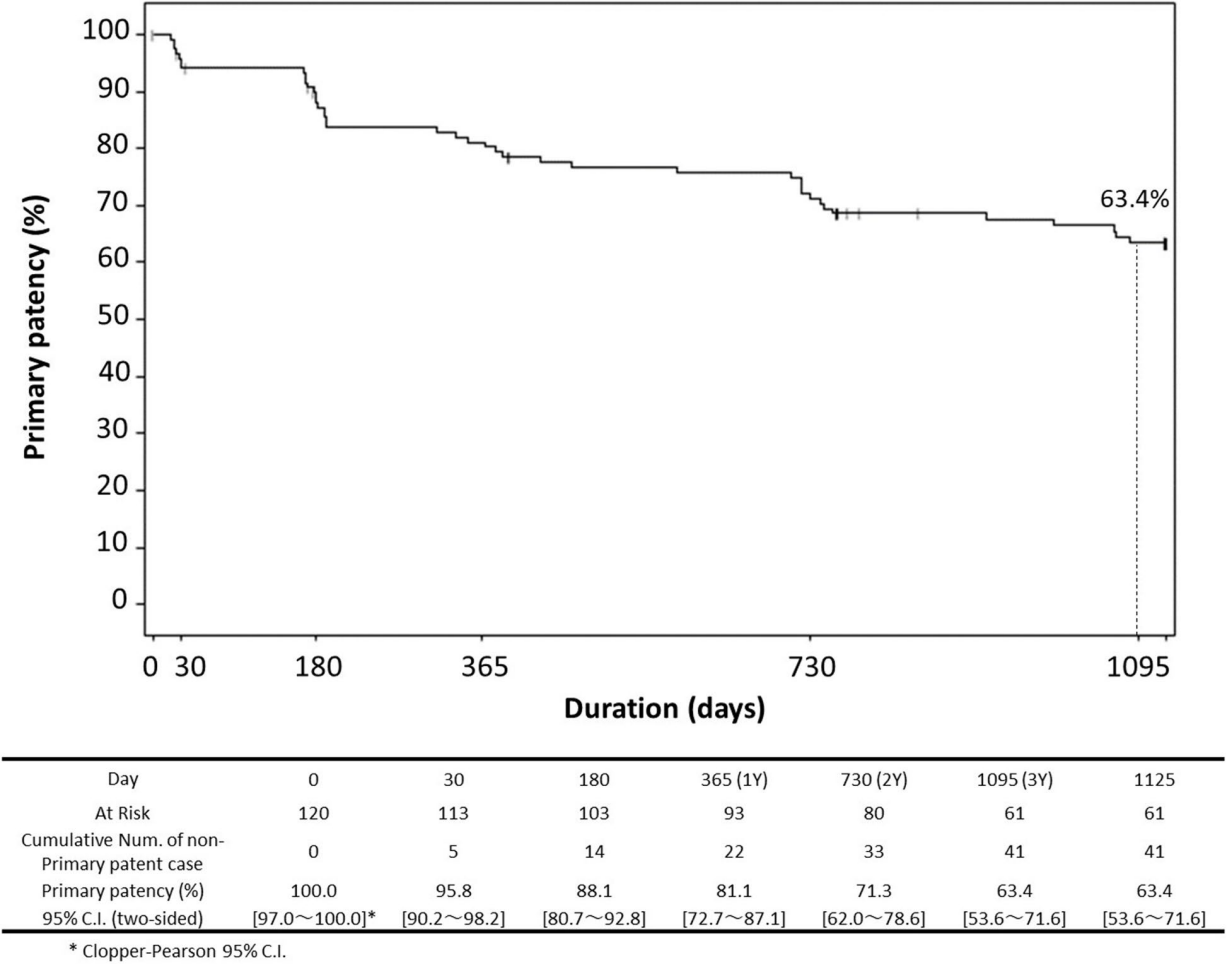
Kaplan–Meier curve for primary patency at 3 years

**Fig. 2:**
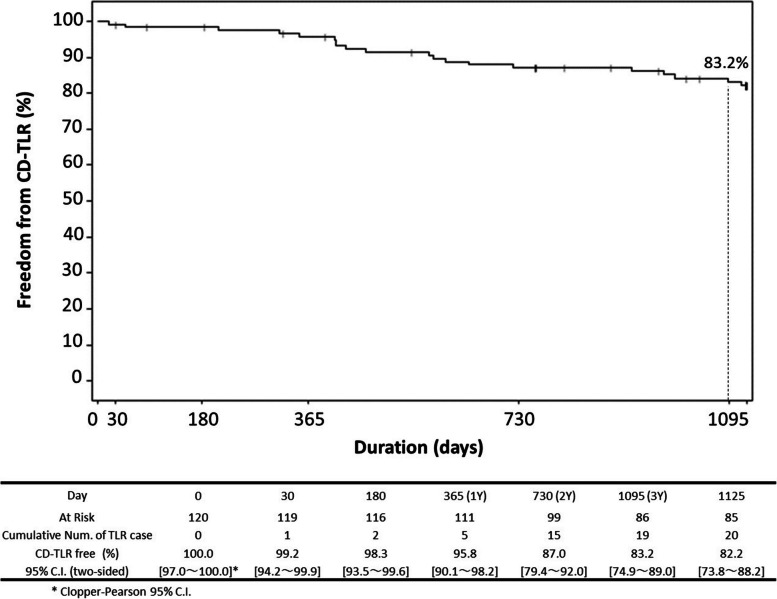
Kaplan–Meier curve for freedom from CD-TLR at 3 years

Of the 121 patients enrolled in this study, one was excluded due to violation of the inclusion criteria, and 120 were included in the analysis. During the 3-year follow-up period, 41 cases of restenosis and 20 cases of TLR were observed. The primary patency rate (Fig. 1) and CD-TLR-free rate (Fig. 2) at 3 years (1,095 days) were 63.4% and 83.2%, respectively. A multivariate Cox proportional hazard regression analysis was conducted to explore predictive factors for the loss of primary patency over a 3-year period (Table 1). The analysis suggested several significant factors, including kidney disease (adjusted hazard ratio [HR]: 0.34, 95% confidence interval [CI]: 0.13–0.88, *p* = 0.027), coronary artery disease (adjusted HR: 0.41, 95% CI: 0.21–0.80, *p* = 0.008), Rutherford class (2, 3 versus 4) (adjusted HR: 7.88, 95% CI: 1.58–39.26, *p* = 0.012), and restenotic lesions (non-stented) (adjusted HR: 2.30, 95% CI: 1.01–5.25, *p* = 0. 048). RVD (≥ 5 mm) that was significant in the univariate analysis did not remain in the multivariate analysis using the backward elimination method with an elimination criterion of a *p* value ≤ 0.1.

**Table 1:** Cox regression at 3 years

Characteristics	Hazard ratio [95% confidence intervals] (*p* value)	
	**Un-adjusted**	**Adjusted**^**c**^
***Clinical characteristics***		
Age (≥ 75 years)	0.76 [0.41, 1.41] (*p* = 0.383)	-
Female	0.95 [0.48, 1.90] (*p* = 0.894)	-
Treatment history of heart disease	0.54 [0.28, 1.04] (*p* = 0.066)	-
Treatment history of lower extremity arterial disease	1.65 [0.85, 3.19] (*p* = 0.136)	-
Diabetes mellitus	1.12 [0.58, 2.16] (*p* = 0.742)	-
Hypertension	0.53 [0.26, 1.08] (*p* = 0.080)	-
Dyslipidemia	2.34 [0.72, 7.58] (*p* = 0.156)	3.18 [0.92, 11.04] (*p* = 0.068)
Kidney disease	0.38 [0.15, 0.97] (*p* = 0.043)	0.34 [0.13, 0.88] (*p* = 0.027)
Heart disease	0.50 [0.26, 0.97] (*p* = 0.039)	-
Coronary artery disease	0.47 [0.25, 0.90] (*p* = 0.021)	0.41 [0.21, 0.80] (*p* = 0.008)
Rutherford Class (2, 3 versus 4)	2.12 [0.51, 8.76] (*p* = 0.302)	7.88 [1.58, 39.26] (*p* = 0.012)
***Angiographic characteristics***		
Restenotic (non-stented)^a^	2.12 [0.94, 4.81] (*p* = 0.071)	2.30 [1.01, 5.25] (*p* = 0.048)
Lesion Length (≥ 150 mm)^b^	1.26 [0.64, 2.47] (*p* = 0.504)	-
PACSS calcification (0, 1, 2 versus 3, 4)^b^	1.21 [0.65, 2.25] (*p* = 0.540)	-
Chronic total occlusion^b^	1.78 [0.87, 3.62] (*p* = 0.115)	-
RVD (≥ 5 mm)^b^	0.49 [0.27, 0.92] (*p* = 0.025)	-
Presence of dissection at the end of index procedure^b^	0.74 [0.38, 1.43] (*p* = 0.368)	-
Final diameter stenosis (> 30%)^b^	0.70 [0.36, 1.37] (*p* = 0.300)	-
Minimum DCB diameter < RVD	0.65 [0.30, 1.41] (*p* = 0.275)	-

*DCB* drug-coated balloon, *PACSS* peripheral arterial calcium-scoring system, *RVD* reference vessel diameter, *TASC* Trans-Atlantic Inter-Society Consensus

^a^Site-reported

^b^Analyzed based on the results of the core laboratory

^c^Adjusted analysis with the use of backward elimination, with an elimination criterion of a *p* value of more than 0.1

**Fig. 3:**
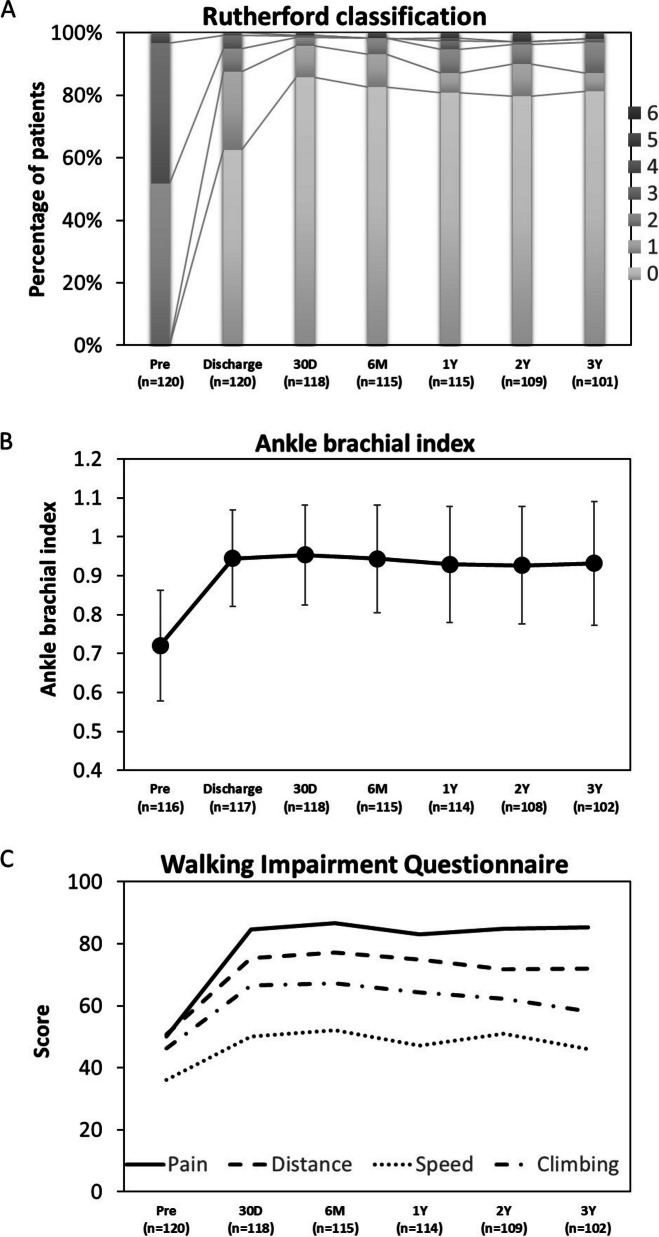
Changes in Rutherford classification, ankle brachial index, and walking impairment questionnaire score over 3 years

The alterations in the Rutherford classification, ABI, and WIQ score are demonstrated in Fig. 3. The enhancements in these quality-of-life indicators were maintained during the 3-year period.

### Safety outcomes

The safety outcomes are detailed in Table 2. Safety evaluation was conducted on all 121 enrolled participants (including one patient who violated the inclusion criteria). Over the 3-year period, the MAE rate was 17.4% (21/121), consisting solely of CD-TLR. No cases of major amputation to the target limb or mortality within 30 days were observed. During the 3-year follow-up period, six patients died. These included two from hepatocellular carcinoma, one each from pneumonia, cerebral contusion, primary lung cancer, and ischemic heart disease. All deaths were determined to be unrelated to the study device and procedure by DSMB.

**Table 2:** Safety outcomes

	**0-30D (*n* = 121)**	**30D-6 M (*n* = 121)**	**6 M-1Y (*n* = 119)**	**1Y-2Y (*n* = 117)**	**2Y-3Y (*n* = 114)**	**Accumulation (*n* = 121)**
**MAE**^a^**, %**	1.7 (2)	0.8 (1)	2.5 (3)	8.5 (10)	4.4 (5)	**17.4 (21)**
Death^b^, %	0 (0)	-	-	-	-	**0 (0)**
CD-TLR, %	1.7 (2)	0.8 (1)	2.5 (3)	8.5 (10)	4.4 (5)	**17.4 (21)**
Amputation^c^, %	0 (0)	0 (0)	0 (0)	0 (0)	0 (0)	**0 (0)**
**All-cause death, %**	0 (0)	0.8 (1)	1.7 (2)	0 (0)	2.6 (3)	**5.0**^**d**^** (6)**

Variables are given as the percentage (counts)

^a^Major adverse event

^b^Device or procedure related death within 30 days after index procedure

^c^Amputation made above the metatarsal line

^d^All deaths were unrelated to the study device and procedure

## Discussion

This study presents the final report of the Kanshas DCB Japan preapproval trial, which evaluated the safety and effectiveness of the device over the 3-year period in Japanese participants with femoropopliteal disease. Although statistical comparisons were not possible, the study population tended to have a higher prevalence of diabetes (67.5%), a higher frequency of bilateral calcification (PACSS grades 3 and 4) (50.8%), and a longer mean lesion length (106.0 mm) than IN.PACT SFA [4], LEVANT 2 [5], and RANGER II SFA [6]. Furthermore, to more closely resemble actual clinical practice, this study allowed the enrollment of non-stented restenotic lesions. In fact, such lesions accounted for 10.8% of the total study lesions, while these anatomical features were identified as significant factors contributing to the loss of primary patency following EVT [7–9]. As discussed later, given that multivariate analysis suggested that restenotic lesions were associated with decreased primary patency, the 3-year primary patency and freedom from CD-TLR observed in this study were similar to those observed with approved DCBs and were considered acceptable. An intriguing observation was that, within the present cohort, the decline in primary patency beyond the first year, as estimated by the Kaplan–Meier method, appeared relatively modest over time. Following the implementation of treatment, enhancements in ABI, WIQ scores, and Rutherford classification were detected and sustained at the 3-year follow-up.

The 3-year MAE rate was 17.4%, consisting solely of CD-TLR, with no instances of target limb major amputation or 30-day mortality. The six fatalities that transpired during the follow-up period were found to be unrelated to the study device and procedure. Over the course of the 3-year period, no safety concerns were identified. These results suggest that the Kanshas DCB can be used effectively and safely over an extended period.

DCB therapy for femoropopliteal artery lesions has demonstrated long-term clinical efficacy and is strongly recommended in current guidelines [10, 11]. First-generation high-dose DCBs provide effective drug retention in the vessel wall but may increase the risk of downstream effects. Experimental studies have shown an association between high-dose DCBs and fibrinoid necrosis in peripheral tissues, along with increased embolic material [12]; however, mortality risk related to paclitaxel devices does not correlate with drug dose [13], and paclitaxel-induced slow-flow phenomena are not linked to worsening chronic limb-threatening ischemia [14]. The result of the PROSPECT MONSTER study [15] in real-world target Japanese populations with complex clinical backgrounds was also similar to the findings of the COMPARE RCT [16], which reported comparable clinical benefits between high-dose and low-dose DCBs despite differences in coating characteristics, highlighting the importance of excipients and coating technology in addition to drug dose. These findings underscore the need for a balanced DCB system that ensures efficient drug transfer and sustained retention within the vessel wall. Kanshas DCB employs the “Unicoat” technology, uniformly coating the balloon surface with paclitaxel microcrystals (3.2 μg/mm^2^) and the carrier L-SEE to enhance arterial drug uptake while minimizing downstream impact [1]. In this study, the Kanshas DCB, classified as a high-dose DCB, exhibited sustained outcomes over a 3-year period without any escalated risk of adverse events, including downstream effects, as determined by the core laboratory.

Exploratory multivariate analysis of variables associated with loss of primary patency suggested that, among baseline characteristics, the presence of restenotic lesions (without stent placement) and Rutherford classification 4 may be independently associated with primary patency loss. An exploratory analysis of the primary patency rate by group of de novo lesions and restenotic lesions using the Kaplan–Meier method (Figure S1) showed a tendency for the patency rate to be lower in the restenotic lesions, although this was not significant. These findings are consistent with the results obtained in other DCB studies [4, 6]. Notably, CTO and RVD (defined as a diameter of less than 5 mm) emerged as an anatomical factor contributing to loss of primary patency at the 2-year mark; however, this association did not reach statistical significance at the 3-year follow-up. In addition, other lesion factors previously identified as positive predictors, including lesion length, severity of vessel calcification, and TASC II classification, were also not statistically significant at the 3-year. During the 3-year period, 41 cases of primary patency loss were documented, with 14 patients (34%) experiencing loss of primary patency at the 6-month mark. The occurrence of target lesion restenosis at the six-month mark may be predominantly ascribed to post-angioplasty angiographic characteristics, such as substantial recoil and vessel dissection, rather than any consequence of the DCB [17]. Huang et al. analyzed 164 patients (mean lesion length 20.4 cm) who underwent DCB treatment and reported that there was a “bimodal pattern” in the timing of restenosis, with diffuse/obstructive restenosis occurring early (median 225 days) and long lesion length being an independent risk factor (*p* = 0.049), while focal restenosis occurred late (median 484 days) and had a good prognosis [18]. In addition, Soga et al. investigated 3165 lesions (new and restenotic) treated with IN.PACT or Lutonix DCB in the POPCORN registry and reported that the independent predictors of restenosis within 1 year were a history of repeat revascularization, small vessel diameter, calcification, CTO, low-dose DCB, and residual stenosis [9].

When these factors were evaluated in our study, we found that the proportion of patients with a residual diameter stenosis of 30% or more was significantly higher in cases with early restenosis (61.5% vs. 17.9%, *p* = 0.0102). The mean residual diameter stenosis rate of early restenosis lesions was 33.3%. It is possible that residual diameter stenosis influenced early restenosis. Due to the limited sample size of this trial, these findings should be interpreted as hypothesis-generating. Confirmation in real-world practice is required. Multivariate analysis suggested that the presence of kidney disease and coronary artery disease functioned as protective factors for primary patency. This discrepancy may be attributable to the instability of results due to the limited sample size and the rigorous outpatient follow-up treatment with anti-atherosclerotic medications. However, the underlying causes of this phenomenon could not be identified in this study. Further investigation is necessary to elucidate the association between DCB failure and the presence of coronary artery disease.

In regard to the safety outcomes, the 3-year evaluation period revealed no occurrence of fatalities or major amputations associated with the study device or procedure, thereby substantiating the safety of the device.

In previous DCB trials, the occurrence of CD-TLR has shown a linear increase each year [19–21]. In the present study, as adjudicated by the CEC, CD-TLR occurred in 20 patients over the 3-year follow-up period, with only four patients requiring reintervention between the second and third years. While this temporal pattern suggests that the absence of CD-TLR within the first 2 years may be associated with a lower likelihood of subsequent CD-TLR, this observation should be interpreted with caution given the limited number of late events.

The apparent attenuation of CD‑TLR beyond 2 years may partly reflect censoring effects, loss to follow-up, a reduced number of patients at risk, and competing risks such as death. Nevertheless, the Kaplan–Meier curve observed 1 year or later after treatment with the Kanshas DCB resembles that historically reported following conventional balloon angioplasty. This finding may indicate a potential unique feature of the Kanshas DCB; however, it should be regarded as hypothesis-generating and warrants confirmation in larger studies with longer follow-up.

## Conclusions

The Kanshas DCB showed favorable safety and effectiveness for treating atherosclerotic lesions in the superficial femoral and proximal popliteal arteries over 3 years.

## Supplementary Information

Supplementary Material 1.

Supplementary Material 2.

## Data Availability

The data supporting the findings of this study are available from Terumo Corporation, Tokyo, Japan, but restrictions apply to the availability of these data, which were used under license for the current study and are not publicly available. Data are available from the authors upon reasonable request and with permission from Terumo Corporation.
