# Supplementary material for: Three-year outcomes of the TCD-17187 (Kanshas) drug-coated balloon for the treatment of atherosclerotic lesions in the superficial femoral and proximal popliteal artery
Source: CVIR Endovasc. 2026 Apr 24;9:48. doi: 10.1186/s42155-026-00695-7 (PMC13109471; doi:10.1186/s42155-026-00695-7)
Supplement: Supplementary file 2 — Supplementary Material 2. [file 42155_2026_695_MOESM2_ESM.docx]

**Supplementary material**

The list of Institutional Review Board (IRB)

1. Chikamori Hospital, Kochi, Japan
2. Kokura Memorial Hospital, Fukuoka, Japan
3. Morinomiya Hospital, Osaka, Japan
4. SUBARU Health Insurance Ota Memorial Hospital, Gunma, Japan
5. Kasukabe Chuo General Hospital, Saitama, Japan
6. Tokyo Bay Medical Center, Chiba, Japan
7. Tokai University Hospital, Kanagawa, Japan
8. Osaka Saiseikai Nakatsu Hospital, Osaka, Japan
9. School of Medicine, Hyogo Medical University, Hyogo, Japan
10. Tokeidai Memorial Hospital, Hokkaido, Japan
11. Toho University Ohashi Medical Center, Tokyo, Japan
12. Showa University School of Medicine, Tokyo, Japan
13. Tokushukai Group Institutional Review Board, Tokyo, Japan*
14. Miyazaki Medical Association Hospital, Miyazaki, Japan
15. Sendai Kousei Hospital, Miyagi, Japan
16. Asahi General Hospital, Chiba, Japan
17. Saiseikai Yokohama City Eastern Hospital, Kanagawa, Japan
18. Jikei University School of Medicine, Tokyo, Japan
19. Tokushima Red Cross Hospital, Tokushima, Japan
20. Kansai Rosai Hospital, Hyogo, Japan

*: Joint IRB.

Table S1 Baseline characteristics and Lesion characteristics

| **Characteristics^a^** | **All (n=120)** |
| --- | --- |
| ***Clinical Characteristics*** |  |
| Age (Years) | 74.5 ± 7.3 |
| Male, % (N) | 73.3 (88) |
| Body mass index (kg/m²) | 23.7 ± 3.7 |
| Hypertension, % (N) | 83.3 (100) |
| Dyslipidemia, % (N) | 84.2 (101) |
| Diabetes mellitus, % (N) | 67.5 (81) |
| Insulin dependent diabetes mellitus, % (N) | 19.2 (23) |
| Kidney disease^b^, % (N) | 24.2 (29) |
| Heart failure, % (N) | 5.8 (7) |
| Coronary artery disease^c^, % (N) | 51.7 (62) |
| Cerebrovascular disease, % (N) | 15.8 (19) |
| Smoking status, % (N) |  |
| Never | 17.5 (21) |
| Past | 53.3 (64) |
| Current | 29.2 (35) |
| Treatment history of coronary artery disease, % (N) | 40.0 (48) |
| Treatment history of cerebrovascular disease, % (N) | 5.0 (6) |
| Treatment history of lower extremity arterial disease, % (N) | 58.3 (70) |
| Endovascular therapy, % (N) | 57.5 (69) |
| Surgical therapy, % (N) | 0.8 (1) |
| ABI before index procedure | 0.72 ± 0.14 |
| Rutherford Class, % (N) |  |
| 2 | 51.7 (62) |
| 3 | 45.0 (54) |
| 4 | 3.3 (4) |
| ***Angiographic Characteristics*** |  |
| De novo, % (N)^d^ | 89.2 (107) |
| Restenotic (non-stented), % (N)^d^ | 10.8 (13) |
| Popliteal Involvement, % (N)^e^ | 1.7 (2) |
| PACSS calcification, % (N)^e^ |  |
| Grade 0 | 39.2 (47) |
| Grade 1 | 8.3 (10) |
| Grade 2 | 1.7 (2) |
| Grade 3 | 31.7 (38) |
| Grade 4 | 19.2 (23) |
| Lesion Length (mm)^e, f^ | 106.0 ± 52.6 |
| Chronic total occlusions, % (N)^e^ | 17.5 (21) |
| TASC II Classification, % (N)^e^ |  |
| A | 42.5 (51) |
| B | 32.5 (39) |
| C | 22.5 (27) |
| D | 2.5 (3) |
| RVD (mm)^e^ | 5.2 ± 0.8 |
| MLD (mm)^e^ | 1.1 ± 0.8 |
| Diameter stenosis (%)^e^ | 78.3 ± 13.8 |
| ABI, Ankle brachial pressure index; BTK, Below the knee; MLD, Minimal lumen diameter; N, numbers in category; n, number of available values; PACSS, Peripheral arterial calcium scoring system; RVD, Reference vessel diameter; TASC, Trans-Atlantic Inter-Society Consensus.  ^a^ Continuous variables are presented as the means ± standard deviation; categorical variables are given as the percentage (counts).  ^b^ Includes: chronic renal failure, glomerulonephritis, diabetic nephropathy, and others.  ^c^ Includes: ischemic heart disease, chronic heart failure, arrhythmia, valvular disease, and others.  ^d^ Site-reported.  ^e^ Per lesion assessment evaluated by the core laboratory.  ^f^ Normal-to-normal by core laboratory quantitative vascular analysis. | |

Table S2 Procedural Characteristics

| **Procedural Characteristics^a^** | **All (n=120)** |
| --- | --- |
| Pre-dilatation, % (N)^b^ | 100 (120) |
| Balloon size of pre-dilatation (mm)^b^ | 5.5 ± 0.8 |
| MLD after pre-dilatation (mm)^c^ | 3.5 ± 0.8 |
| Diameter stenosis after pre-dilatation (%)^c^ | 31.8 ± 12.4 |
| Dissection^e^ after pre-dilatation, % (N)^c^ |  |
| None | 28.3 (34) |
| A-C | 70.8 (85) |
| D-F | 0.8 (1) |
| DCB size (mm) | 5.7 ± 0.8 |
| Transition time of DCB proceeding (sec)^b^ | 26.4 ± 16.9 |
| Inflation time of DCB dilatation (sec)^b^ | 205.3 ± 48.1 |
| Inflation pressure of DCB (atm)^b^ | 9.0 ± 2.0 |
| The ratio of DCB size/reference vessel diameter^c^ | 1.11 ± 0.14 |
| MLD after DCB dilatation (mm)^c^ | 3.8 ± 0.8 |
| Diameter stenosis after DCB dilatation (%)^c^ | 27.8 ± 10.9 |
| Dissection^e^ after DCB dilatation, % (N)^c^ |  |
| None | 26.7 (32) |
| A-C | 70.0 (84) |
| D-F | 2.5 (3) |
| NA | 0.8 (1) |
| Post-dilatation, % (N) | 9.2 (11) |
| Dissection length after DCB dilatation (mm)^c^ | 29.3 ± 20.5 |
| Provisional stenting rate, % (N)^b^ | 0.8 (1) |
| Final diameter stenosis (>30%), % (N)^c^ | 37.5 (45) |
| Achievement of TIMI 3 flow grade, % (N)^c, d^ | 97.5 (117) |
| Number of BTK run off^c^ | 1.4 ± 0.9 |
| Technical Success, % (N) | 100.0 (120) |
| Procedural Success, % (N) | 100.0 (120) |
| BTK, Below the knee; DCB, Drug-coated balloon; MLD, Minimal lumen diameter; N, numbers in category; n, number of available values; NA, Not applicable; TIMI, Thrombolysis in Myocardial Infarction Grade.  ^a^ Continuous variables are presented as the means ± standard deviation; categorical variables are given as the count/sample (percentage).  ^b^ Site-reported.  ^c^ Per lesion assessment evaluated by the core laboratory.  ^d^ TIMI grade flow 3 indicates complete perfusion.  ^e^ Dissection grades were evaluated by Peripheral Arterial Calcium Scoring System (PACSS). | |

Figure S1 Kaplan-Meier curves for primary patency rates of de novo and restenotic lesions at 3 years.


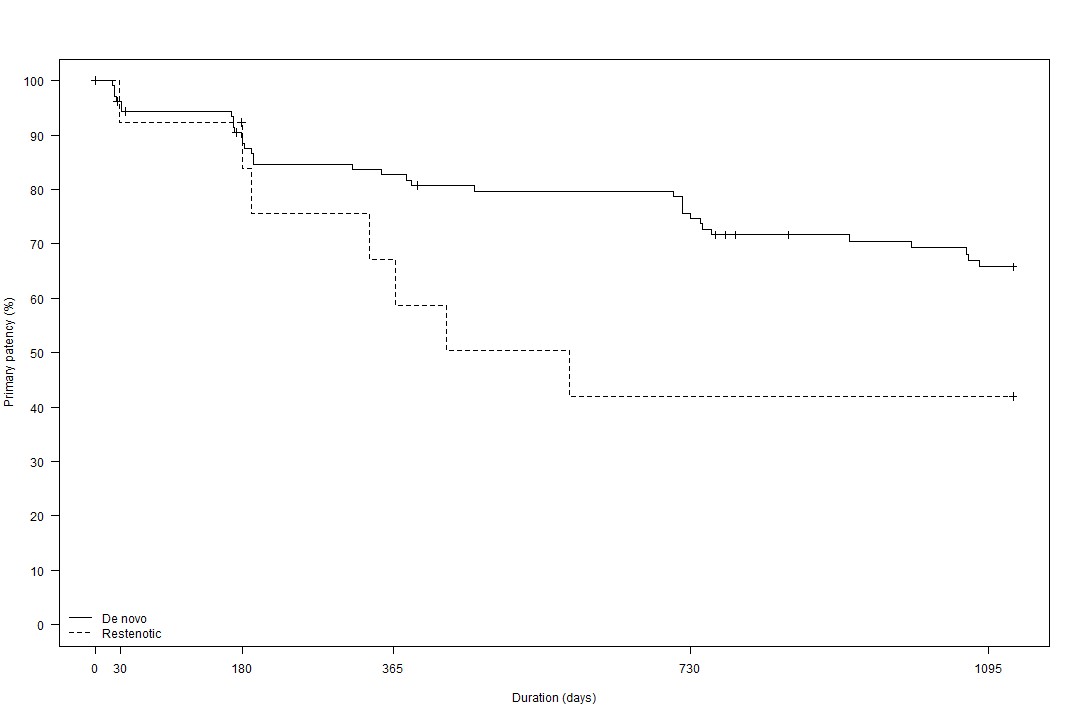


| Day | | 0 | 30 | 180 | 365 (1Y) | 730 (2Y) | 1095 (3Y) |
| --- | --- | --- | --- | --- | --- | --- | --- |
| De novo | |  |  |  |  |  |  |
|  | At risk | 107 | 101 | 92 | 85 | 75 | 56 |
|  | Cumulative Num. of non-Primary patent case | 0 | 4 | 12 | 18 | 26 | 34 |
|  | Primary patent (%) | 100 | 96.2 | 88.5 | 82.7 | 74.7 | 65.8 |
|  | 95% C.I. (two-sided) | [96.6, 100.0]* | [90.3, 98.6] | [80.7, 93.3] | [73.9, 88.7] | [65.1, 82.0] | [55.5, 74.3] |
| Restenotic | |  |  |  |  |  |  |
|  | At risk | 13 | 12 | 11 | 8 | 5 | 5 |
|  | Cumulative Num. of non-Primary patent case | 0 | 1 | 2 | 4 | 7 | 7 |
|  | Primary patent (%) | 100 | 92.3 | 83.9 | 67.1 | 42.0 | 42.0 |
|  | 95% C.I. (two-sided) | [75.3, 100.0]* | [56.6, 98.9] | [49.4, 95.7] | [34.2, 86.2] | [15.4, 66.8] | [15.4, 66.8] |
| * Clopper-Pearson confidence interval | | |  |  |  |  |  |
